# Supplementary material for: AMaze: an intuitive benchmark generator for fast prototyping of generalizable agents
Source: Front Artif Intell. 2025 Mar 26;8:1511712. doi: 10.3389/frai.2025.1511712 (PMC11979106; doi:10.3389/frai.2025.1511712)
Supplement: Supplementary file 1 [file Data_Sheet_1.pdf]

Supplementary Material

1 INTERPOLATION TRAINING DETAILS

1.1 Mazes

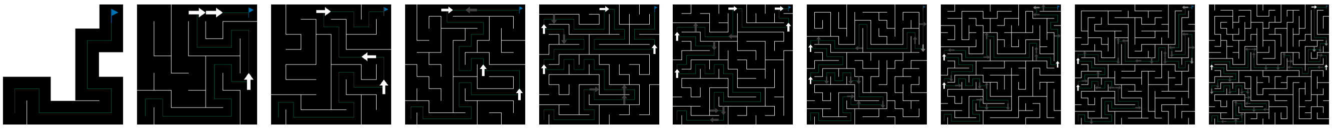

a: Training

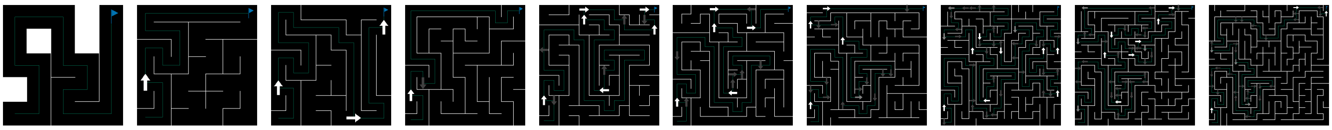

b: Evaluation

Figure S1. Mazes used in the interpolation training (top) and for intermediate evaluations (bottom).

Stage	Training	Evaluation
0	M2000007_5x5_U_C1	M2000018_5x5_U_C1
1	M2000007_7x7_C1	M2000018_7x7_C1
2	M2000007_8x8_C1	M2000018_8x8_C1
3	M2000007_10x10_C1_l.028_L.25	M2000018_10x10_C1_l.028_L.25
4	M2000007_12x12_C1_l.065_L.25	M2000018_12x12_C1_l.065_L.25
5	M2000007_13x13_C1_l.1_L.25_t.056_T.5	M2000018_13x13_C1_l.1_L.25_t.056_T.5
6	M2000007_15x15_C1_l.14_L.25_t.17_T.5	M2000018_15x15_C1_l.14_L.25_t.17_T.5
7	M2000007_17x17_C1_l.18_L.25_t.28_T.5	M2000018_17x17_C1_l.18_L.25_t.28_T.5
8	M2000007_18x18_C1_l.21_L.25_t.39_T.5	M2000018_18x18_C1_l.21_L.25_t.39_T.5
9	M2000007_20x20_C1_l.25_L.25_t.5_T.5	M2000018_20x20_C1_l.25_L.25_t.5_T.5

Table S1. Textual representation of the mazes used for the interpolation training and intermediate evaluations. These can be used with the amaze library by invoking it as `amaze --maze <string>`

2 AGENTS TRAINING BEHAVIORS

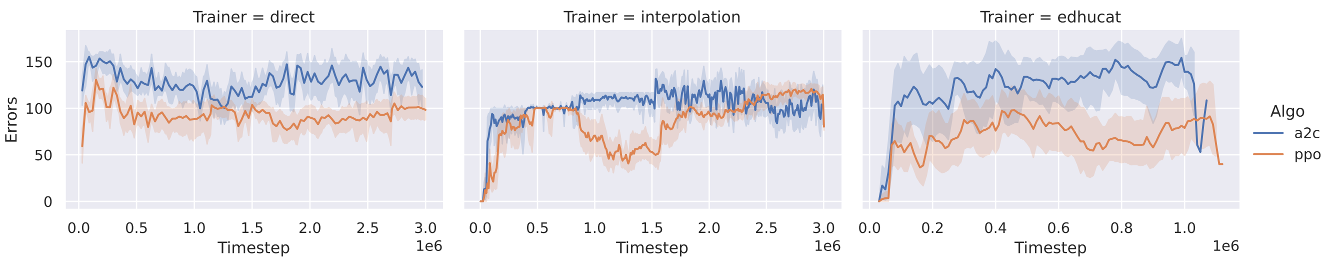

Figure 2a. Aggregated errors

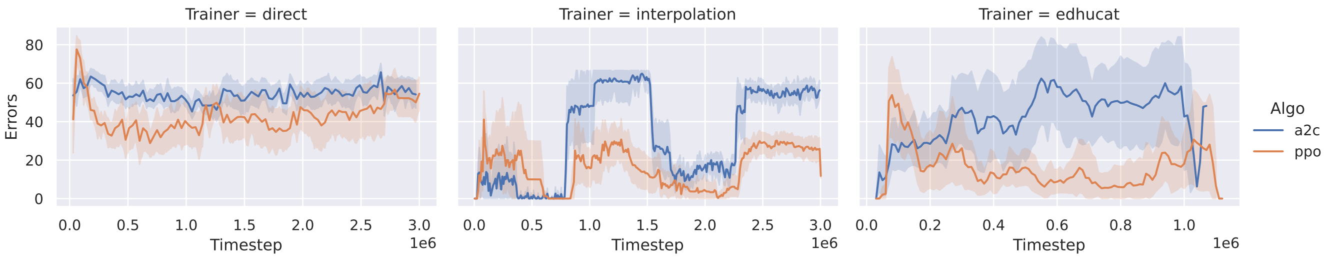

Figure 2b. Errors on Clues

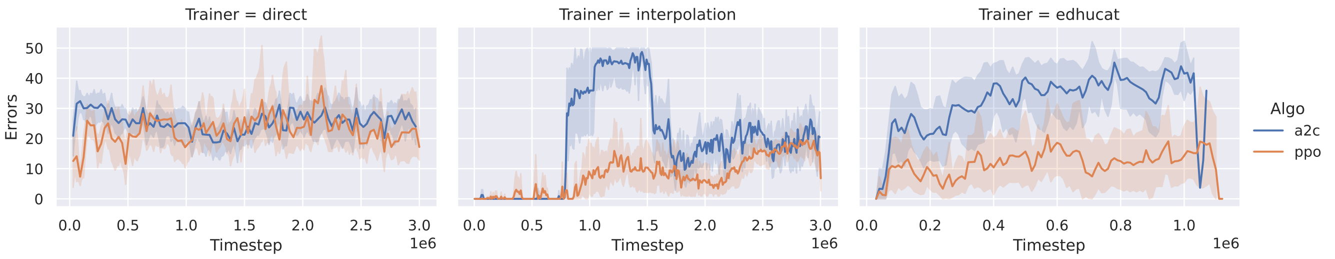

Figure 2c. Errors on Lures

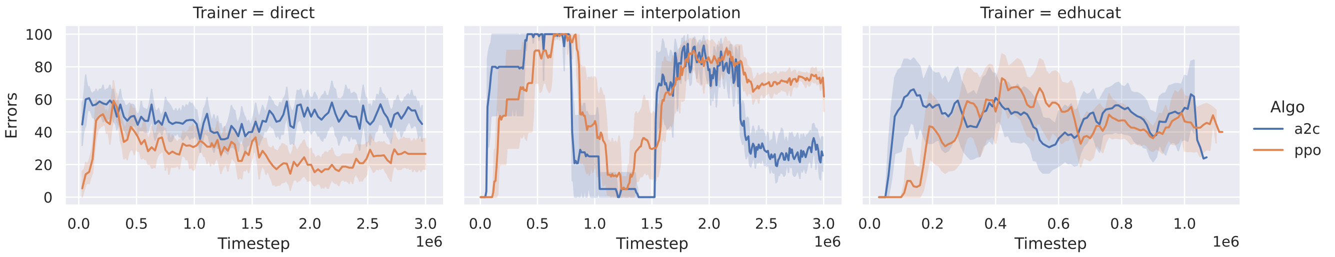

Figure 2d. Errors on Traps

Figure 2. Details of the agent's errors during the training under each regimes (left to right) and with both algorithms (A2C and PPO). Globally, the direct trainer shows relatively high variance. In both other cases, changing maze results in drastic changes in error rate. These are more quickly compensated with PPO except for *Lures*. As a whole, PPO is again found to generally perform better than A2C. Note that, while using the same budget *globally*, agents trained with EDHuCAT have only continuously learned for about 1/3 of that time.
